# Efficacy and effectiveness of Ceftaroline Fosamil in patients with pneumonia: a systematic review and meta-analysis

**DOI:** 10.1186/s12931-018-0905-x

**Published:** 2018-10-23

**Authors:** Giovanni Sotgiu, Stefano Aliberti, Andrea Gramegna, Marco Mantero, Marta Di Pasquale, Federica Trogu, Laura Saderi, Francesco Blasi

**Affiliations:** 1 Clinical Epidemiology and Medical Statistics Unit, Department of Clinical and Experimental Medicine, University of Sassari, Sassari, Italy; 2 Department of Pathophysiology and Transplantation, University of Milan, Milan, Italy; 3 Internal Medicine Department, Respiratory Unit and Cystic Fibrosis Adult Center, Fondazione IRCCS Ca’ Granda Ospedale Maggiore Policlinico, Milan, Italy

**Keywords:** CAP, HAP, VAP, HCAP, MRSA, Stewardship, Safety

## Abstract

**Background:**

Pneumonia is a relevant clinical and public health issue worldwide frequently associated with infections caused by Multi-Drug Resistant (MDR) pathogens. Ceftaroline fosamil is a promising new antibiotics with broad-spectrum bacterial activity. The aim of this systematic review and meta-analysis is to assess the efficacy and the effectiveness of ceftaroline fosamil in community-acquired (CAP), hospital-acquired (HAP), healthcare-associated (HCAP) and ventilator-associated (VAP) pneumonia.

**Methods:**

A systematic review and meta-analysis was carried out retrieving both experimental and observational studies.

**Results:**

A total of 2364 records was found and 14 manuscripts were finally considered eligible. The pooled efficacy/effectiveness was 81.2% (I^2^: 1.2%) in all types of pneumonia. The pooled relative risk of clinical cure was 1.1 (I^2^: 0.0%). The success rate was higher than 70% for infections caused by *S. pneumoniae* and *S. aureus*, including MDR pathogens.

**Conclusions:**

Ceftaroline fosamil showed a high efficacy/effectiveness in patients with any type of pneumonia with a good safety profile.

**Electronic supplementary material:**

The online version of this article (10.1186/s12931-018-0905-x) contains supplementary material, which is available to authorized users.

## Background

Pneumonia is one of the major threats and leading cause of death due to infectious diseases worldwide, in both adults and children [1]. Mortality for community-acquired pneumonia (CAP) ranges from < 5% among outpatients up to 30% in those admitted in an intensive care unit [2]. Hospital-acquired pneumonia is the second most common nosocomial infection and the first in terms of mortality [3]. One of the major drivers of the high impact of pneumonia on patients’ morbidity and mortality is represented by infections with multi-drug resistant (MDR) bacteria and, among them, methicillin resistant *Staphylococcus aureus* (MRSA) plays a relevant rule in both CAP and HAP [4].

Over the past two decades, antimicrobial resistance has become a tangible reality not only for patients with hospital-acquired (HAP), ventilator-associated (VAP) or healthcare-associated (HCAP) pneumonia but also for those coming from the community [5]. It has been recognized as a clinical and public health threat, which should be immediately addressed in order to avoid a dramatic back to a pre-antibiotic era. The clinical mismanagement of the antibiotics and the misuse in agriculture and in veterinary medicine are favoring the rapid increase of the rate of antibiotic resistant bacterial strains worldwide. The research and development activities of the pharmaceutical companies in the bacterial field have significantly decreased since the 1980s’ for several reasons, including an increased prevalence of chronic diseases, the complex design of the clinical trials requested by regulatory agencies, and the increasing antibiotic resistance rates.

One of the most promising antibiotics recently marketed is ceftaroline fosamil, a fifth-generation cephalosporin which proved a both in vitro and in vivo broad-spectrum activity against gram-positive (including methicillin susceptible and resistant *S. aureus* -MSSA and MRSA) and –negative bacteria. Ceftaroline fosamil showed clinical and bacteriological efficacy against bacterial pathogens responsible of CAP and skin infections [6]. Furthermore, during the pre-marketing studies, it showed a good safety and tolerability profile [7].

The aim of the present study was to perform a systematic review and meta-analysis to evaluate the efficacy and the effectiveness of ceftaroline fosamil in patients with any kind of pneumonia described in experimental and observational studies, respectively.

## Methods

### Search strategy

9pt?>Experimental and observational studies aimed to evaluate the efficacy/effectiveness of ceftaroline fosamil in adult hospitalized patients and outpatients with a diagnosis of pneumonia, including CAP, HAP, VAP, and HCAP, were selected. The search was conducted in three electronic databases: PubMed, Scopus, and Cochrane Central Register of Controlled Trials, without any time restrictions. Only publications written in English language were selected. Several key-words, combined using different strings according to the electronic database protocols, were used: “Ceftaroline”, “Ceftaroline Fosamil”, and “Respiratory Infections”. To increase the search sensitivity, the list of references of the selected articles, as well as published systematic or narrative reviews, were manually and carefully assessed to include manuscripts not cited in the search record lists. Abstracts of the main pulmonology, infectious diseases, or microbiology conferences were not searched based on the poor information they could provide on the selection criteria and on the main clinical and bacteriological findings. Furthermore, non-peer-reviewed articles of the grey literature were not considered based on their poor clinical and methodological reliability.

### Article selection

Only articles clearly describing the primary objective of this systematic review, i.e. efficacy/effectiveness of Ceftaroline fosamil in patients with pneumonia (CAP, HAP, VAP, and HCAP) were selected. At least one of the following efficacy/effectiveness-related outcomes were considered: 1) number of responders/number of subjects in group at day 4 after initiating therapy; 2) cure rate at the end of therapy (EOT); 3) cure rate at the test of cure (8–15 days after the end of therapy, TOC); 4) 14-day clinical success/cure (±1 day) from the diagnosis of pneumonia. The assessment of the outcomes and, then, the suitability of the article was carried out during the evaluation of the abstract or the full-text article if the information was not stated in the abstract. Studies were included if adult (≥18 years of age) patients, recruited in the ceftaroline fosamil or in the control arm, were at least 20.

The following exclusion criteria were adopted for the searched records: 1) papers written in languages other than English; 2) narrative or systematic reviews and meta-analyses; 3) abstracts presented in national and international conferences; 4) editorials, research letters, commentaries, correspondences; 5) case-reports or –series; 6) manuscripts focused only on efficacy/effectiveness of ceftaroline fosamil in infections other than respiratory.

Safety and tolerability profile of ceftaroline fosamil included only the collection of the adverse events.

Records were independently assessed by two researchers (LS and FT). They carefully evaluated titles and contents of the abstracts. In case of potential interesting articles, they retrieved and assessed the full-text. Inconsistencies during the first and second phase were solved by a third and senior reviewer (GS), who supervised the entire selection and review process.

### Data extraction

Qualitative and quantitative data were extracted by the same reviewers (LS and FT) who selected the articles. Collection of the variables was decided during the preparation of the study protocol, as well as the implementation of an ad-hoc standardized form in an Excel format (Microsoft Office). Disparity during data collection was solved by a third reviewer (GS) by consensus. However, the final inter-rater agreement was approximately 100%. A random cross-check was carried out for ~ 20% of the selected citations.

The following variables were collected: response rate at day 4, response rate EOT, response rate TOC, 14-day clinical cure, publication year, epidemiological study design, country/ies where the study was carried out, study period, sample size (total, ceftaroline and control arm), age, gender, ethnic origin, severity of pneumonia, including the Pneumonia Severity Index (PSI) [2], lobar infiltration, pleural effusion, parenchymal or airway disease, previous episodes of pneumonia or bacteremia, previous exposure to antibiotics, asthma, etiology (i.e., *S. pneumoniae*, MSSA and MRSA), and adverse events. According to the Italian law on epidemiological studies based on anonymous and aggregated data, no ethical clearance was requested to the ethical committees of Milan and Sassari, Italy.

### Study quality assessment

The systematic review and meta-analysis was carried out following the Preferred Reporting Items for Systematic Reviews and Meta-Analyses (PRISMA) [8]. No significant inconsistencies and disparities were detected in the selection and data extraction phases. The agreement between LS and FT was higher than 97% and incongruences were solved by the intervention of GS.

### Statistical analysis

A descriptive analysis of qualitative and quantitative variables was performed using proportions and central tendency/variability indicators (i.e., mean and standard deviation, SD), respectively. A meta-analysis was conducted for the efficacy/effectiveness-related outcomes. Forest plots were adopted to show the characteristics (i.e., between-study variability and sample size) of the single outcomes in comparison with the pooled estimates. Point and interval (95% confidence intervals, CI) estimates were used for studies’ and pooled outcomes. The inconsistency (I^2^) indicator was computed to show the variability across studies and was statistically tested with the chi-squared test for heterogeneity. Fixed or random-effects models were implemented according to the assumption that the true effect is or is not the same in all studies, respectively. Stratified analyses were conducted following the type of pneumonia (i.e., CAP, HAP, VAP, HCAP) or the etiology (i.e., *S. pneumoniae*, MSSA and MRSA). A two-tailed *p*-value less than 0.05 was considered statistically significant. The statistical software STATA version 14 (STATACorp LP, College Station, TX 77845, USA) was used to perform all statistical computations.

## Results

### Selection of the studies

**Fig. 1:**
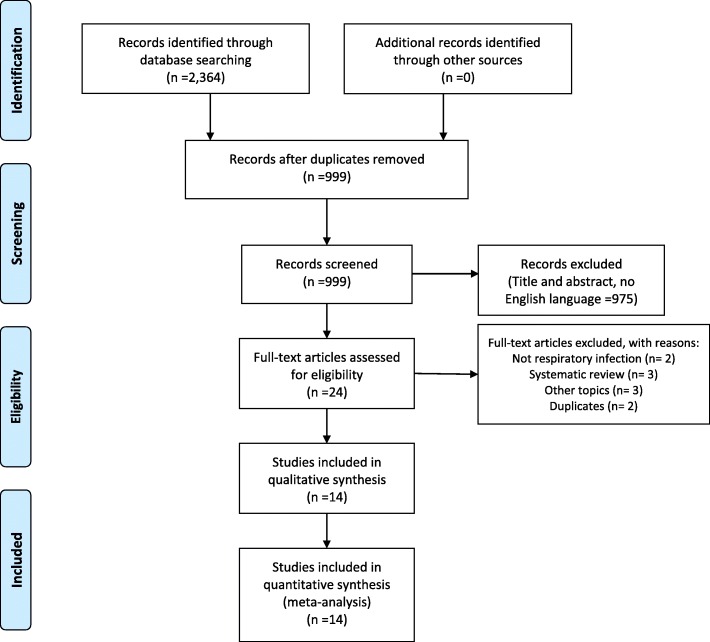
Flow-chart of the systematic review

The search of the three electronic databases found 2364 records (Fig. 1). After the removal of duplicates, 999 citations were screened and only 14 were considered suitable for a qualitative and quantitative analysis.

### Characteristics of the selected studies

Six [9–14] (42.9%) studies were clinical trials, published in the time period 2010–2015, and 7 [15–21] (50.0%) were observational retrospective studies (6, 85.7%, cohort studies and 1, 14.3%, case-control study), published between 2014 and 2016 (Table 1). Only one [22] (7.1%) study was a retrospective analysis of previous clinical trials. In the majority of the cases (13/14, 92.9%), studies were carried out from 2007 to 2014 in USA; only the clinical trial of Zhong et al. [14] was conducted in five Asian countries. The efficacy/effectiveness of the ceftaroline fosamil arm was compared with that of a control group in 8 (57.1%) studies [9–15, 22]; in the remaining 6 studies [16–21] no comparators were chosen. The dosage of ceftaroline fosamil was the same across all 14 studies [9–22] (i.e., 600 mg every 12 h). The ceftriaxone was the most frequently (7/8, 87.5%) prescribed antibiotic in controlled studies (Additional file 1: Table S1).

**Table 1:** Summary of the selected studies

First Author	Title	Publication year	Type of study	Country	Study period
Jandourek et al. [9]	Efficacy of ceftaroline fosamil for bacteremia associated with community-acquired bacterial pneumonia	2014	Clinical Trial	USA	Jul 2007-Dec 2008
File et al. [10]	FOCUS 1: a randomized, double-blinded, multicentre, Phase III trial of the efficacy and safety of ceftaroline fosamil versus ceftriaxone in community-acquired pneumonia	2011	Clinical Trial	USA	Jan 2008-Dec 2008
Low et al. [11]	FOCUS 2: a randomized, double-blinded, multicentre, Phase III trial of the efficacy and safety of ceftaroline fosamil versus ceftriaxone in community-acquired pneumonia	2011	Clinical Trial	USA	Jul 2007-Aug 2008
Shorr et al. [12]	Assessment of ceftaroline fosamil in the treatment of community-acquired bacterial pneumonia due to Streptococcus pneumoniae: insights from two randomized trials	2013	Clinical Trial	USA	Jul 2007-Dec 2008
File et al. [13]	Integrated analysis of FOCUS 1 and FOCUS 2: randomized, doubled-blinded, multicenter phase 3 trials of the efficacy and safety of ceftaroline fosamil versus. Ceftriaxone in patients with community-acquired pneumonia	2010	Clinical Trial	USA	Jul 2007-Dec 2008
Zhong et al. [14]	Ceftaroline fosamil versus ceftriaxone for the treatment of Asian patients with community-acquired pneumonia: a randomised, controlled, double-blind, phase 3, non-inferiority with nested superiority trial	2015	Clinical Trial	China, India, South Korea, Taiwan, Vietnam	Dec 2011-Apr 2013
Arshad et al. [15]	Ceftaroline fosamil for treatment of Methicillin-Resistant *Staphylococcus aureus* hospital-acquired pneumonia and health care-associated pneumonia. A 5-year matched case-control evaluation of epidemiology and outcomes	2016	Case-control study	USA	Jan 2009-May 2013
Eckburg et al. [22]	Day 4 Clinical response of ceftaroline fosamil versus ceftriaxone for community-acquired bacterial pneumonia	2012	Retrospective integrated analysis of FOCUS trials	USA	Jul 2007-Dec 2008
Guervil et al. [16]	Ceftaroline fosamil as first-line versus second-line treatment for acute bacterial skin and skin structure infections (ABSSSI) or community-acquired bacterial pneumonia (CABP)	2015	Retrospective Cohort study	USA	Aug 2011-Feb 2013
Udeani et al... [17]	Ceftaroline fosamil for the treatment of community-acquired bacterial pneumonia in elderly patients	2014	Retrospective Cohort study	USA	Aug 2011-Ap 2013
Ramani et al..... [18]	Contemporary use of ceftaroline fosamil for the treatment of community-acquired bacterial pneumonia: CAPTURE study experienc	2014	Retrospective Cohort study	USA	Aug 2011-Feb 2013
Vasquez et al... [19]	Ceftaroline Fosamil for the Treatment of Staphylococcus aureus Bacteremia Secondary to Acute Bacterial Skin and Skin Structure Infections or Community-Acquired Bacterial Pneumonia	2015	Retrospective Cohort study	USA	Aug 2011-Feb 2013
Casapao et al... [20]	Large retrospective study evaluation of the effectiveness and safety of Ceftaroline fosamil therapy	2014	Retrospective observational study	USA	Jan 2011-Jun 2013
Kaye et al. [21]	Ceftaroline fosamil for the treatment of hospital acquired pneumonia and ventilator associated pneumonia	2015	Retrospective Cohort study	USA	Sep 2013-Mar 2014

### Characteristics of the enrolled cohort compared with a control group

The sample size of the studies with a control arm ranged from 45 to 1153 patients; in particular, the ceftaroline fosamil arm ranged from 23 to 580 patients, whereas the control arm sized from 22 to 573 (Table 2). The mean (SD) age of the ceftaroline and the control group was ~ 60 (15) years, ranging from 58.8 (16.1) to 66.1 (14.7) years and from 58.8 (16.4) to 65.8 (13.9) years, respectively. The proportion of males was higher than 60%, both in the ceftaroline and in the control arm, in all studies, with the only exception of that of Arshad et al. [15], where the percentage of males was ~ 50% in both treatment groups. Three (37.5%) studies [9, 12, 22] did not describe the ethnic origin of the cohort; 3 studies [10, 11, 15] showed a highest (> 80%) prevalence of white patients in both arms, whereas the studies of Zhong et al. [14] and Arshad et al. [15] had a high proportion of Asian and black patients, respectively.

**Table 2:** Demographics by treatment groups

Study	Sample size, n	Sample size, n		Mean (SD) age, y		Male, n (%)		Ethnic origin^a^, n (%)	
		Ceftaroline group	Control group	Ceftaroline group	Control group	Ceftaroline group	Control group	Ceftaroline group	Control group
*Jandourek* et al.*..., 2014* [9]	45	23	22	60.6 (16.1)	63.2 (16.2)	15 (65.2)	17 (77.3)	–	–
*File* et al.*., 2011* [10]	591	291	300	61.0 (16.6)	61.2 (16.4)	187 (64.3)	191 (63.7)	260 (89.3)	268 (83.3)
*Low* et al*, 2011* [11]	562	289	273	60.6 (16.1)	62.0 (14.7)	175 (60.6)	175 (64.1)	278 (96.2)	264 (96.7)
*Shorr* et al.*.., 2013* [12]	139	69	70	63 (17)	62 (15)	43 (62.3)	47 (67.1)	–	–
*File* et al.*, 2010* [13]	1153	580	573	60.8 (16.4)	61.6 (15.6)	362 (62.4)	366 (63.9)	538 (92.8)	532 (92.8)
*Zhong* et al*, 2015* [14]	763	381	382	66.1 (14.7)	65.8 (13.9)	265 (69.6)	272 (71.2)	381 (100.0) A	382 (100.0) A
*Arshad* et al.*..., 2016* [15]	149	40	109	58.8 (16.1)	58.8 (16.4)	20 (50.0)	54 (49.5)	16 (40.0) B	46 (42.2) B
*Eckburg* et al.*., 2012* [22]	309	154	155	59.9 (17.7)	60.5 (16.0)	99 (64.3)	97 (62.6)	–	–

^a^Proportion of white patients, unless otherwise specified as black (B), or Asian (A)

The PSI was heterogeneous (Table 3); the majority of the patients were diagnosed as risk class III or IV. Only the study of Arshad et al. [15] did not provide a pneumonia severity classification. Half of the patients in the ceftaroline fosamil and control arm were diagnosed as PSI risk class III. However, the study of Jandourek et al. [9] recruited two third of the patients diagnosed as PSI risk class IV. A description of a multi-lobar infiltration, as well as of a pleural effusion, was performed by two (25.0%) studies [9, 12]. At least one chronic parenchymal or airway disease (including COPD, bronchiectasis, and interstitial fibrosis) affected a proportion of patients ranging from 20.0 to 33.2% per single arm. Asthma was described only by four (50.0%) studies [10, 11, 13, 14] and was found in less than 10% of the patients. Previous episodes of pneumonia were recorded in one fifth of the treatment group; however, this information was provided only by three (37.5%) studies [10, 11, 13]. Four (50.0%) studies [10–12, 22] found that 1/3–1/2 of the cohort was previously treated with antibiotics. The prevalence of bacteremia was very low (< 10%) per single arm in 4 (50.0%) studies [10, 11, 13, 14]. Only the study of Jandourek et al. [9] found a bacteremia prevalence of 100%.

**Table 3:** Clinical baseline characteristics by treatment groups

Study	Ceftaroline group	Control group	Ceftaroline group	Control group	Ceftaroline group	Control group	Ceftaroline group	Control group	Ceftaroline group	Control group
	PSI risk class III, n (%)		PSI risk class IV, n (%)		Severe CAP^a^, n (%)		Multilobar infiltrate, n (%)		Pleural effusion, n (%)	
*Jandourek* et al.*., 2014* [9]	5 (21.7)	5 (22.7)	18 (78.3)	15 (68.2)	–	–	5 (21.7)	5 (22.7)	5 (21.7)	5 (22.7)
*File* et al.*......., 2011* [10]	190 (65.3)	182 (60.7)	101 (34.7)	118 (39.3)	82 (28.2)	89 (29.7)	–	–	–	–
*Low* et al.*..., 2011* [11]	170 (58.8)	171 (62.6)	119 (41.2)	102 (37.4)	99 (34.3)	80 (29.3)	–	–	–	–
*Shorr* et al.*........., 2013* [12]	34 (49.3)	37 (52.9)	35 (50.7)	33 (47.1)	22 (31.9)	32 (45.7)	18 (26.1)	21 (30.0)	15 (21.7)	13 (18.6)
*File* et al*, 2010* [13]	360 (62.1)	353 (61.6)	220 (37.9)	220 (38.4)	–	–	–	–	–	–
*Zhong* et al.*., 2015* [14]	255 (67.0)	265(69.4)	126 (33.1)	117 (30.6)	–	–	–	–	–	–
*Arshad* et al*, 2016* [15]	–	–	–	–	–	–	–	–	–	–
*Eckburg* et al*, 2012* [22]	84 (54.5)	82 (52.9)	61 (39.6)	61 (39.4)	–	–	–	–	–	–
	Structural lung disease^b^, n (%)		Prior pneumonia, n (%)		Asthma, n (%)		Prior antimicrobial therapy, n (%)		Bacteremia, n (%)	
*Jandourek* et al*, 2014* [9]	–	–	–	–	–	–	–	–	23 (100.0)	22 (100.0)
*File* et al*, 2011* [10]	64 (22.0)	60 (20.0)	61 (21.0)	51 (17.0)	25 (8.6)	25 (8.3)	137 (47.1)	143 (47.7)	8 (2.7)	9 (3.0)
*Low* et al*, 2011* [11]	96 (33.2)	87 (31.9)	62 (21.5)	41 (15.0)	24 (8.3)	13 (4.8)	100 (34.6)	117 (42.9)	15 (5.2)	11 (4.0)
*Shorr* et al*, 2013* [12]	–	–	–	–	–	–	26 (37.7)	32 (45.7)	19 (27.5)	13 (18.6)
*File* et al*, 2010* [13]	160 (27.6)	147 (25.7)	123 (21.2)	92 (16.1)	49 (8.4)	38 (6.6)	–	–	23 (4.0)	20 (3.5)
*Zhong* et al*, 2015* [14]	120 (31.5)^c^	121 (31.7)^c^	–	–	21 (5.5)	22 (5.8)	80 (21.0)	85 (22.3)	3 (0.8)	5 (1.3)
*Arshad* et al*, 2016* [15]	8 (20.0)	31 (28.4)	–	–	–	–	4 (11.8)	24 (23.3)	–	–
*Eckburg* et al*, 2012* [22]	43 (27.9)	41 (26.5)	–	–	–	–	57 (37.0)	68 (43.9)	23 (14.9)	21 (13.5)
	Diabetes mellitus, n (%)									
*Jandourek* et al*, 2014*	–	–								
*File* et al*, 2011*	–	–								
*Low* et al*, 2011*	–	–								
*Shorr* et al*, 2013*	–	–								
*File* et al*, 2010*	–	–								
*Zhong* et al*, 2015*	62 (16.3)	62 (16.3)								
*Arshad* et al.*, 2016*	10 (25.0)	20 (18.4)								
*Eckburg* et al.*.., 2012*	–	–								

^a^Modified ATS severe CAP criteria include the presence of three or more of the following at baseline: respiratory rate ≥ 30 breaths/min; O2,90% or PaO2,60 mmHg; multilobar infiltrates; confusion/disorientation; blood urea nitrogen level ≥ 20 mg/dL; leucopenia (WBC count,4000 cells/mm3); thrombocytopenia (platelet count,100,000 cells/mm3); hypothermia (core temperature,368C); systolic blood pressure,90 mmHg; or diastolic blood pressure ≤ 60 mmHg. (*Niederman MS, Mandell LA, Anzuetto A* et al.*........... Guidelines for the management of adults with community-acquired pneumonia: diagnosis, assessment of severity, antimicrobial therapy, and prevention. Am J Respir Crit Care Med 2001; 163: 1730–54*)

^b^Defined as any chronic parenchymal or airway disease [e.g. chronic obstructive pulmonary disease (emphysema, chronic bronchitis), bronchiectasis, or interstitial fibrosis]

^c^only COPD and chronic bronchitis

### Characteristics of the enrolled cohort without a control group

Six [16–21] out fourteen (42.9%) studies evaluated the efficacy/effectiveness of ceftaroline fosamil without a control arm. The sample size was heterogeneous, from 21 to 528 patients (Table 4). The mean (SD) age was high, ranging from 60 (18) to 64.3 (1.7) years. The proportion of males ranged from 48.3 to 57.5%.

**Table 4:** Demographic characteristics of subjects treated with only ceftaroline

Study	Sample size, n	Mean (SD) age, y	Male, n (%)
*Guervil* et al.*.., 2015* [16]	396	64.3 (1.7)	198 (50.0)
*Udeani* et al.*., 2014* [17]	528	63.6 (20.2)	255 (48.3)
*Ramani* et al*, 2014* [18]	398	63.5 (17.8)	199 (50.0)
*Vasquez* et al.*, 2015* [19]	21	60 (18)	11 (52.4)
*Casapao* et al.*., 2014* [20]	92	–	–
*Kaye* et al*, 2015* [21]	40	61.3 (16.8)	23 (57.5)

The prevalence of patients with chronic parenchymal or airway diseases was significantly higher if compared with that found in the cohort of patients recruited in controlled studies (40.7–47.5%) (Table 5). Furthermore, in three out four studies (75%) [16–18] one fifth of those treated with ceftaroline fosamil suffered of congestive heart failure. However, the percentage of previous episodes of pneumonia was similar (range: 24.6–25.4%). Three (50.0%) studies [16, 18, 19] described previous antimicrobial exposure, which was higher than 82%.

**Table 5:** Clinical characteristics of subjects treated with only ceftaroline

Study	Structural lung disease^a^, n (%)	Congestive heart failure, n (%)	Prior pneumonia, n (%)	GERD, n (%)	Smoking, n (%)	Prior antimicrobial therapy, n (%)
*Guervil* et al*, 2015* [16]	161 (40.7)	79 (20.0)	98 (24.8)	91 (23.0)	114 (28.8)	396 (100.0)
*Udeani* et al.*...., 2014* [17]	228 (43.2)	113 (21.4)	134 (25.4)	127 (24.1)	159 (30.1)	–
*Ramani* et al.*., 2014* [18]	162 (40.7)	80 (20.1)	98 (24.6)	92 (23.1)	114 (28.6)	328 (82.4)
*Vasquez* et al.*.., 2015* [19]	–	–	–	–	–	18 (85.7)
*Casapao* et al.*....., 2014* [20]	–	–	–	–	–	–
*Kaye* et al.*, 2015* [21]	19 (47.5)	8 (0.20)	10 (25.0)	10 (25.0)	21 (52.5)	–

^a^Defined as any chronic parenchymal or airway disease [e.g. chronic obstructive pulmonary disease (emphysema, chronic bronchitis), bronchiectasis, or interstitial fibrosis]

### Efficacy and effectiveness of ceftaroline fosamil

**Fig. 2:**
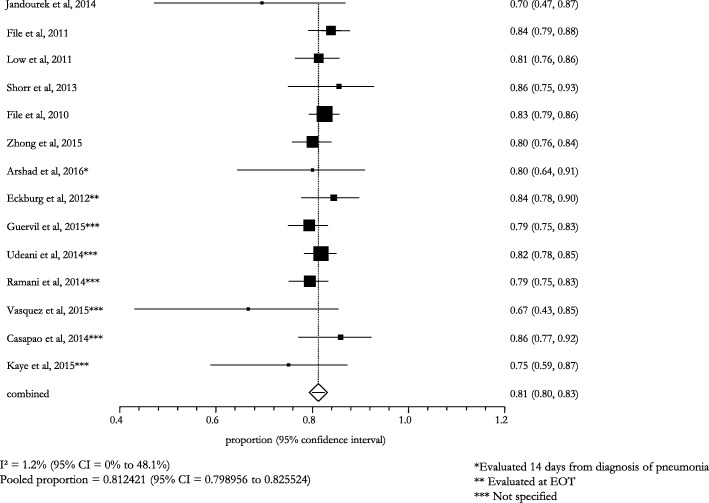
Efficacy of ceftaroline in the overall pneumonia (including CAP, VAP, HCAP, HAP)

**Fig. 3:**
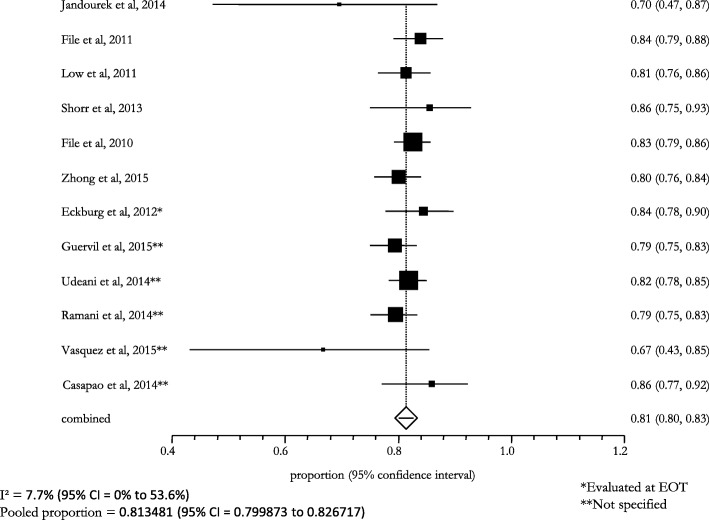
Clinical success in CAP subjects treated with ceftaroline

**Fig. 4:**
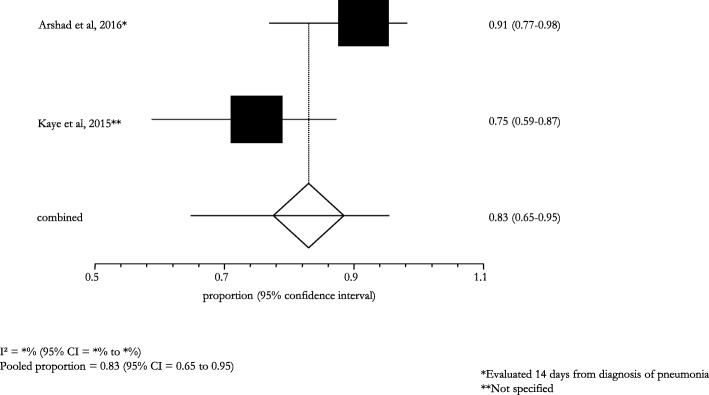
Clinical success in HAP/VAP/HCAP subjects treated with ceftaroline

The overall efficacy/effectiveness of ceftaroline fosamil in all types of pneumonia (i.e., CAP, VAP, HCAP, HAP) cases was 81.2% (95% CI: 79.9–82.6; I^2^: 1.2%) (Fig. 2). The pooled treatment success rate was equal to 81.3% (95% CI: 80.0–82.7; I^2^: 7.7%) in patients with CAP, recruited in 12 (85.7%) studies (Fig. 3); a similar pooled clinical success (83.0%, 95% CI: 65.0–95.0; I^2^: -) was found for patients with VAP, HCAP, and HAP, enrolled in 2 (14.3%) studies (Fig. 4).

**Fig. 5:**
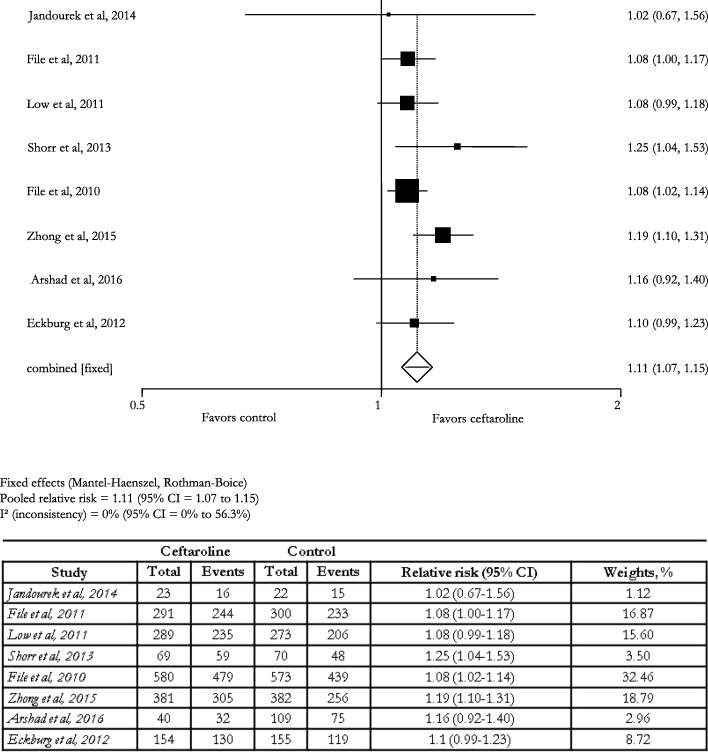
Effect of ceftaroline on clinical cure

In the 8 (57.1%) controlled studies [9–15, 22] the pooled relative risk of clinical cure was 1.1 (95% CI: 1.1–1.2; I^2^: 0.0%) (Fig. 5).

**Fig. 6:**
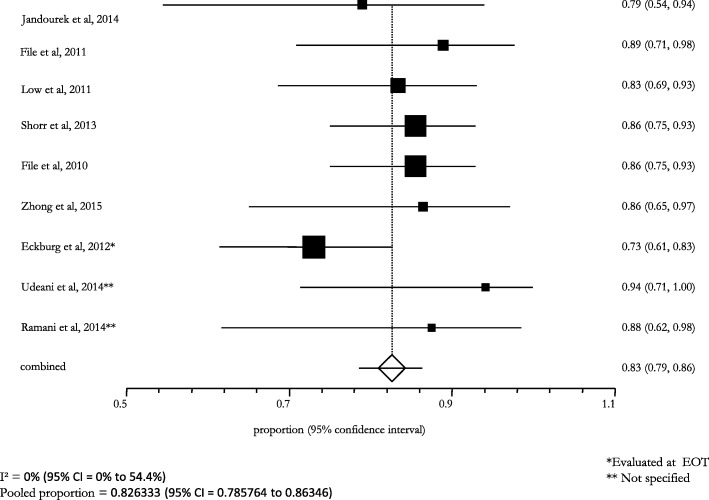
Clinical and microbiological response rates in subjects with *Streptococcus pneumoniae* treated with ceftaroline

**Fig. 7:**
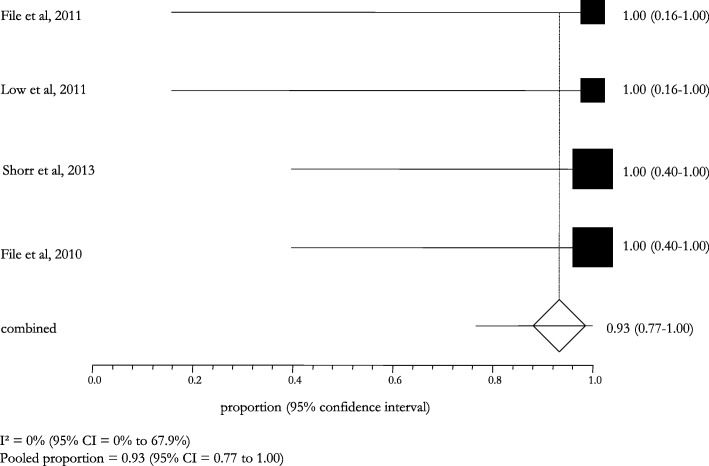
Clinical cure rates in subjects with *MDR Streptococcus pneumoniae* treated with ceftaroline at TOC visit

**Fig. 8:**
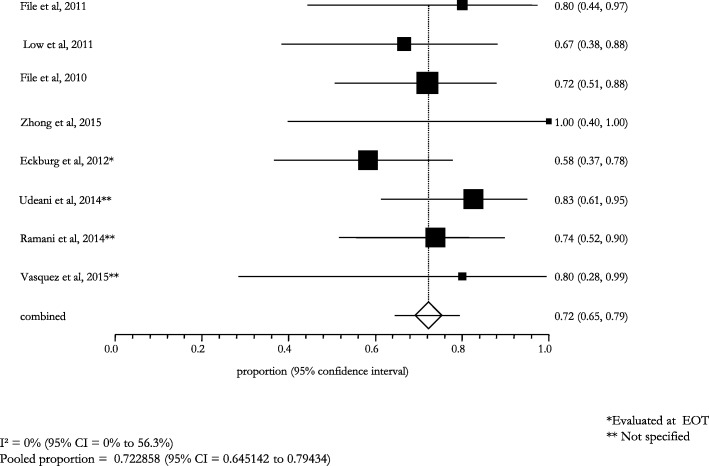
Clinical and microbiological response rates in subjects with *MSSA* treated with ceftaroline

**Fig. 9:**
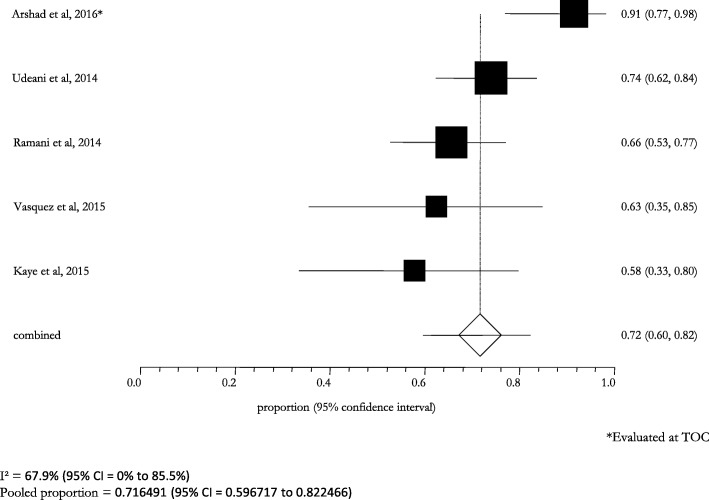
Clinical and microbiological response rates in subjects with *MRSA* treated with ceftaroline

The stratified analysis according to the microbiological diagnosis showed a high clinical and microbiological success: it was 82.6% (95% CI: 78.6–86.4; I^2^: 0.0%) in patients with a lung infection caused by *S. pneumoniae*, 93.0% (95% CI: 77.0–100.0; I^2^: 0.0%) in those with a MDR *S. pneumoniae* infection (Figs. 6 and 7). The cases of pneumonia caused by MSSA and MRSA showed a pooled success rate of 72.3% (95% CI: 64.5–79.4; I^2^: 0.0%) and 71.7% (95% CI: 59.7–82.3; I^2^: 67.9%), respectively (Figs. 8 and 9).

### Safety and tolerability of ceftaroline fosamil

Only 4 (28.6%) studies [10, 11, 13, 14] described the safety profile of the ceftaroline fosamil and control group: the percentage of adverse events ranged from 39.9 to 53.7% in the ceftaroline fosamil arm, whereas it ranged from 42.7 to 47.2% in the control arm (Table 6). The most frequently reported adverse events were: diarrhea, headache, insomnia, nausea, phlebitis, hypertension, and hypokalemia; however, their point prevalence was less than 5% in the ceftaroline fosamil group. Mortality rate in ceftaroline-treated arm is summarized in the Table 7.

**Table 6:** Adverse events (safety population)

Study	Ceftaroline group	Control group	Ceftaroline group	Control group	Ceftaroline group	Control group	Ceftaroline group	Control group
	Any adverse events, n (%)		Diarrhea, n (%)		Headache, n (%)		Insomnia, n (%)	
*File* et al.*..........., 2011* [10]	119/298 (39.9)	136/308 (44.2)	14/298 (4.7)	7/308 (2.3)	10/298 (3.4)	4/308 (1.3)	9/298 (3.0)	6/308 (1.9)
*Low* et al.*, 2011* [11]	196/315 (53.7)	145/307 (47.2)	12/315 (3.8)	9/307 (2.9)	11/315 (3.5)	5/307 (1.6)	10/315 (3.2)	8/307 (2.6)
*File* et al*, 2010* [13]	288/613 (47.0)	281/615 (45.7)	26/613 (4.2)	16/615 (2.6)	21 /613 (3.4)	9/615 (1.5)	19/613 (3.1)	14/615 (2.3)
*Zhong* et al.*.., 2015* [14]	172/381 (45.1)	163/383 (42.7)	24/381 (6.3)	13/383 (3.4)	6/381 (1.6)	9/383 (2.4)	–	–
	Nausea, n (%)		Phlebitis, n (%)		Hypertension, n (%)		Hypokalaemia, n (%)	
*File* et al*, 2011* [10]	8/298 (2.7)	8/308 (2.6)	7/298 (2.3)	5/308 (1.6)	6/298 (2.0)	8/308 (2.6)	4/298 (1.3)	10/308 (3.2)
*Low* et al*, 2011* [11]	6/315 (1.9)	6/307 (2.0)	10/315 (3.2)	8/307 (2.6)	8/315 (2.5)	8/307 (2.6)	10/315 (3.2)	5/307 (1.6)
*File* et al*, 2010* [13]	14/613 (2.3)	14/615 (2.3)	17/613 (2.8)	13/615 (2.1)	14/613 (2.3)	16/615 (2.6)	14/613 (2.3)	15/615 (2.4)
*Zhong* et al*, 2015* [14]	8/381 (2.1)	3/383 (0.8)	–	–	–	–	5/381 (1.3)	4/383 (1.1)

**Table 7:** Mortality rate in the ceftaroline and control groups

Study	Mortality rate, n (%)	
	Ceftaroline group	Control group
*Jandourek* et al*, 2014*	–	–
*File* et al*, 2011*	6/298 (2.0)	6/308 (1.9)
*Low* et al*, 2011*	9/315 (2.9)	6/307 (2.0)
*Shorr* et al*, 2013*	–	1/70 (1.4)
*File* et al*, 2010*	15/613 (2.4)	12/615 (2.0)
*Zhong* et al*, 2015*	3/381 (0.8)	4/383 (1.0)
*Arshad* et al*, 2016 28-day mortality*	4/40 (10.0)^a^	16/109 (14.7)^a^
*Eckburg* et al*, 2012*	–	–
*Ramani* et al*, 2014*	8/398 (2.0)^b^	–
*Casapao* et al*, 2014*	13/92 (14.1)^b^	–
*Vasquez* et al*, 2015*	1/21 (4.8)	–
*Guervil* et al*, 2015*	8/396 (2.0)^b^	–
*Kaye* et al*, 2015*	5/40 (12.5)	–
*Udeani* et al*, 2014*	15/528 (2.8)	–

^a^28-day mortality

^b^ Hospital mortality

## Discussion

This systematic review and meta-analysis confirms the positive results on ceftaroline fosamil described in single observational and experimental studies. It pools observational findings from a real-world scenario as well as experimental results from the clinical trial world. The final message highlights the high efficacy and effectiveness of ceftaroline fosamil in patients with different types of pneumonia, including CAP, HAP, VAP and HCAP, as well as its good safety and tolerability profile.

All the selected studies found a high pooled clinical success rate (> 80%) across all types of pneumonia, demonstrating poor variability between studies and between pneumonia types. However, the majority of the selected studies focused the attention on CAP and only two studies on pneumonias other than CAP [15, 21].

One of the most important findings is the high microbiological cure against drug-susceptible and –resistant *S. pneumoniae* strains. In the last two decades, it has been described the emergence and spread of MDR isolates, as well as the decreased vaccination coverage and the replacement of vaccine-related with other non-vaccine-related serotypes. The possibility of increasing the current antibiotic *armamentarium* with new effective and safe drugs can improve the prognosis of some patients.

More attention needs to be deserved to the MSSA and MRSA. The current therapeutic options (e.g., glycopeptides or linezolid) could be inappropriate or not available in some geographical settings. The high frequency of MRSA both in the hospital and in the community should be carefully monitored and adequate therapeutic options are necessary. Ceftaroline fosamil has demonstrated a high clinical cure rate (> 70%) in forms of pneumonia caused by MSSA and MRSA strains.

The broad-spectrum activity of ceftaroline fosamil against gram-positive and –negative bacteria could represent an added value in case of complicated polymicrobial infections, as well as in case of empirical therapy when the collection of respiratory specimens is negative or not feasible.

Several limitations of the present study can be detected: only experimental studies with positive and significant results on ceftaroline fosamil could have been published, representing a publication bias. Nevertheless, the confirmation of the experimental findings with observational and real-life results supports the reliability of the clinical and microbiological findings. The selection of non-controlled studies could reduce the statistical power of some findings and represent a methodological limitation; however, the necessity of recruiting real-life studies and the homogeneity of the results on the efficacy across the studies provide robustness to the inferential analysis. The geographical representation is partially jeopardized, with a highest prevalence of studies carried out in the USA. This could reduce the generalizability of the findings to some geographical areas (e.g., Africa, Europe), which could show differences in terms of microbial ecology (antibiotic resistance rates and different microbial burden) and of patients’ characteristics. Yet, the scientific evidence provided by high-, middle-, and low-income (Asian countries) settings could reduce this selection bias, as demonstrated by Aliberti et al. [4].

It was proved a clear confirmation of the efficacy and effectiveness of ceftaroline fosamil in patients with CAP but only a few studies (i.e., two) assessed its role in other pneumonia entities (e.g., HCAP), which can be caused by MDR bacteria in difficult-to-treat patients with severe clinical conditions and comorbidities. One of the main shortcomings of the published studies is the limited focus on the antibiotic safety. An individual patient data meta-analysis, with the involvement of all the authors of the published studies, could better analyze this critical point.

No studies have evaluated pharmacological interactions in randomized clinical trials with other antibiotics or drugs prescribed for chronic diseases. Furthermore, evidence should be provided for some at-risk patient categories, such as children, pregnant and breast-feeding women, elderly people. This systematic review selected clinical studies where a high proportion of patients with comorbidities was enrolled. However, more significant findings are needed, along with specific studies on the efficacy against other less incident bacterial pathogens.

In conclusion, this study provides a systematic collection and critical analysis of the present scientific evidence on ceftaroline fosamil. A few years after its distribution in the market, it has been shown its high efficacy and effectiveness, as well as its safety and tolerability. However, its effectiveness can be preserved in the near future if prescribed appropriately following antimicrobial stewardship policies and proved in vitro drug susceptibility in individual cases.

## Additional file

Additional file 1: **Table S1.** Antibiotics prescribed in the control group. **Table S2.** Etiology of the infected patients recruited in the selected studies. (DOCX 17 kb)
