# Supplementary material for: Efficacy and effectiveness of Ceftaroline Fosamil in patients with pneumonia: a systematic review and meta-analysis
Source: Respir Res. 2018 Oct 23;19:205. doi: 10.1186/s12931-018-0905-x (PMC6199731; doi:10.1186/s12931-018-0905-x)
Supplement: Supplementary file 1 — Table S1. Antibiotics prescribed in the control group. Table S2. Etiology of the infected patients recruited in the selected studies. (DOCX 17 kb) [file 12931_2018_905_MOESM1_ESM.docx]

**Table S1. Antibiotics prescribed in the control group**

| **Study** | **Control group** |
| --- | --- |
| *Jandourek et al, 2014* | Ceftriaxone |
| *File et al, 2011* | Ceftriaxone |
| *Low et al, 2011* | Ceftriaxone |
| *Shorr et al, 2013* | Ceftriaxone |
| *File et al, 2010* | Ceftriaxone |
| *Zhong et al, 2015* | Ceftriaxone |
| *Arshad et al, 2016* | Other agents: vancomycin (87%), vancomycin and cefepime (51%), Linezolid (36%) |
| *Eckburg et al, 2012* | Ceftriaxone |

**Table S2. Etiology of the infected patients recruited in the selected studies.**

| **Study** | **Pathogens isolated** | **n/N (%)** |
| --- | --- | --- |
| *Jandourek et al, 2014* | Streptococcus pneumoniae | 31/45 (68.9) |
|  | MDRSP | 2/45 (4.4) |
|  | PSSP | 30/45 (66.7) |
|  | Staphylococcus aureus | 7/45 (15.6) |
|  | MRSA | 1/45 (2.2) |
|  | Escherichia coli | 2/45 (4.4) |
|  | Haemophilus influenzae | 5/45 (11.1) |
|  | Klebsiella pneumoniae | 1/45 (2.2) |
|  | Pseudomonas stutzeri | 1/45 (2.2) |
| *File et al, 2011* | Streptococcus pneumoniae | 57/591 (9.7) |
|  | MDRSP | 3/591 (0.5) |
|  | Staphylococcus aureus | 24/591 (4.1) |
|  | Escherichia coli | 15/591 (2.5) |
|  | Haemophilus parainfluenzae | 18/591 (3.1) |
|  | Klebsiella pneumoniae | 13/591 (2.2) |
|  | Enterobacter cloacae | 14/591 (2.4) |
|  | Enterobacter cloacae | 15/591 (2.5) |
| *Low et al, 2011* | Streptococcus pneumoniae | 82/562 (14.6) |
|  | MDRSP | 10/562 (1.8) |
|  | Staphylococcus aureus | 31/562 (5.5) |
|  | Haemophilus influenzae | 29/562 (5.2) |
|  | Haemophilus parainfluenzae | 17/562 (3.0) |
|  | Klebsiella pneumoniae | 15/562 (2.7) |
|  | Escherichia coli | 10/562 (1.8) |
|  | Enterobacter cloacae | 5/562 (0.9) |
| *Shorr et al, 2013* | Streptococcus pneumoniae | 139/139 (100.0) |
| *File et al, 2010* | Streptococcus pneumoniae | 139/1153 (12.1) |
|  | MDRSP | 13/1153 (1.1) |
|  | Staphylococcus aureus | 75/1153 (6.5) |
|  | MRSA | 2/1153 (0.2) |
|  | Haemophilus influenzae | 44/1153 (3.8) |
|  | Haemophilus parainfluenzae | 35/1153 (3.0) |
|  | Klebsiella pneumoniae | 28/1153 (2.4) |
|  | Klebsiella oxytoca | 25/1153 (2.2) |
| *Zhong et al, 2015* | Streptococcus pneumoniae | 37/119 (31.1) |
|  | Staphylococcus aureus | 8/119 (6.7) |
|  | Klebsiella pneumoniae | 30/119 (25.2) |
|  | Escherichia coli | 9/119 (7.6) |
|  | Haemophilus influenzae | 18/119 (15.1) |
|  | Haemophilus parainfluenzae | 6/119 (5.0) |
| *Arshad et al, 2016* | MRSA | 149/149 (100) |
| *Eckburg et al, 2012* | Streptococcus pneumoniae | 149/309 (48.2) |
|  | Staphylococcus aureus | 55/309 (17.8) |
|  | Streptococcus pyogenes | 1/309 (0.3) |
|  | Haemophilus influenzae | 49/309 (15.9) |
|  | Klebsiella pneumoniae | 30/309 (9.7) |
|  | Klebsiella oxytoca | 12/309 (3.9) |
|  | Escherichia coli | 25/309 (8.1) |
|  | Enterobacter cloacae | 19/309 (6.2) |
|  | Enterobacter aerogenes | 2/309 (0.7) |
|  | Moraxella catarrhalis | 7/309 (2.3) |
|  | Proteus mirabilis | 3/309 (1.0) |
|  | Serratia marcescens | 6/309 (1.9) |
|  | Serratia liquefaciens | 1/309 (0.3) |
|  | Citrobacter koseri | 3/309 (1.0) |
|  | Citrobacter freundii complex | 2/309 (0.7) |
| *Ramani et al, 2014* | MRSA | 25/159 (15.7) |
|  | MSSA | 10/159 (6.3) |
|  | Streptococcus pneumoniae | 6/159 (3.8) |
|  | Escherichia coli | 3/159 (1.9) |
|  | Klebsiella pneumoniae | 3/159 (1.9) |
| *Casapao et al, 2014* | - | - |
| *Vasquez et al, 2015* | MRSA | 16/21 (76) |
| *Guervil et al, 2015* | MRSA | 63/396 (15.9) |
|  | MSSA | 23/396 (5.8) |
|  | Streptococcus pneumoniae | 16/396 (4.0) |
| *Kaye et al, 2015* | MRSA | 19/40 (47.5) |
|  | Escherichia coli | 3/40 (7.5) |
|  | Pseudomonas aeruginosa | 2/40 (5.0) |
|  | Klebsiella oxytoca | 2/40 (5.0) |
|  | MSSA | 1/40 (2.5) |
| *Udeani et al, 2014* | MRSA | 73/528 (13.8) |
|  | MSSA | 23/528 (4.4) |
|  | Streptococcus pneumoniae | 17/528 (3.2) |

MRSA: Methicillin-Resistant Staphylococcus aureus

MSSA: Methicillin-Susceptible Staphylococcus aureus

MDRSP: Multi-Drug Resistant Streptococcus pneumoniae

PSSP: Penicilllin-Susceptible Streptococcus pneumoniae
